# Monstrous Invasion of Placenta Percreta and Previa: Multidisciplinary Management of a Case, the Role of a Urologist, and a Literature Review

**DOI:** 10.7759/cureus.28537

**Published:** 2022-08-29

**Authors:** Gurpremjit Singh, Smrati Sabnani, Shiv C Navriya, Shreya Panda, Amrita Gaurav

**Affiliations:** 1 Urology, All India Institute of Medical Sciences, Rishikesh, Rishikesh, IND; 2 Obstetrics and Gynaecology, All India Institute of Medical Sciences, Rishikesh, Rishikesh, IND; 3 Urology, All India Institute of Medical Sciences, Jodhpur, Jodhpur, IND

**Keywords:** placenta previa, prenatal diagnosis, placenta accreta, postpartum hemorrhage, invasive placenta, morbid adherent placenta

## Abstract

In rare situations, pregnant women may experience life-threatening bleeding due to the placenta’s aberrant invasion of the bladder. A 28-year-old pregnant female with two previous cesarean deliveries presented with the chief complaint of abdominal pain at the earlier scar site. Ultrasound imaging was suggestive of placenta percreta with bladder invasion. The patient underwent elective cesarean section with a uterine-preservation strategy. A healthy male baby was delivered by classical cesarean section, and bilateral uterine artery ligation was done. The patient developed severe postoperative hemorrhage, for which she was re-explored, and the urology team was called for intraoperative assistance. The area of placental invasion into the bladder was resected entirely with bladder reconstruction. Placenta percreta is a life-threatening condition that can involve adjacent uterine structures. Successful management involves a multidisciplinary strategy involving experienced obstetricians, urologists, anesthesiologists, blood bank teams, and neonatologists.

## Introduction

In rare situations, pregnant women may experience life-threatening bleeding as a result of the placenta’s aberrant invasion of the bladder. Retained placental tissues account for 5-10% of postpartum hemorrhages. At the site of placental implantation, a layer of decidua normally separates the placental villi from the uterine myometrium [1]. When the placenta adheres directly to the myometrium without an intervening decidua, it is referred to as placenta accreta, which is one of the causes of retained placental tissue [2,3]. There is a disruption in the endometrial-myometrial interface resulting in a lack of normal decidualization in the area of a previous scar, allowing unusually deep placental anchoring villi and trophoblast infiltration [4]. Placenta accreta is classified into the following three types: placenta accreta vera where the villi attach to the surface myometrium, placenta increta where the villi attach to the myometrium body, and placenta percreta where the villi penetrate the entire thickness of the myometrium [3].

When bladder invasion occurs in the placenta percreta, the mortality rates for mothers are as high as 9.5% and up to 24% for the child. There has been an estimated 50 times increase in the incidence of placenta percreta in the last 50 years [5]. The exact incidence of placenta percreta is difficult to ascertain.

## Case presentation

A 28-year-old lady, G3P2 at 32+5 weeks of gestation, was referred to our center with a diagnosis of placenta previa. The patient was complaining of abdominal pain for five days at the scar site of the previous surgery. She had an obstetric history of two lower uterine cesarean sections, with her last childbirth one year back; therefore, having a short inter-conception period during the current pregnancy. Her other laboratory investigations were suggestive of moderate anemia.

Ultrasound showed placenta previa with loss of myometrial interface and bladder wall invasion, which was suggestive of placenta percreta with fluid collection and echoes in the retroplacental and subchorionic region.

The patient was planned for a uterus-preserving surgery by a multidisciplinary approach involving an obstetrician team, interventional radiologist team, and urology team for bilateral internal iliac artery balloon catheter placement, followed by cesarean delivery and embolization. An emergency laparotomy was performed because of severe scar tenderness. On the day of the surgery, the patient could not undergo catheter placement due to a malfunctioning fluoroscopy suite.

**Figure 1:**
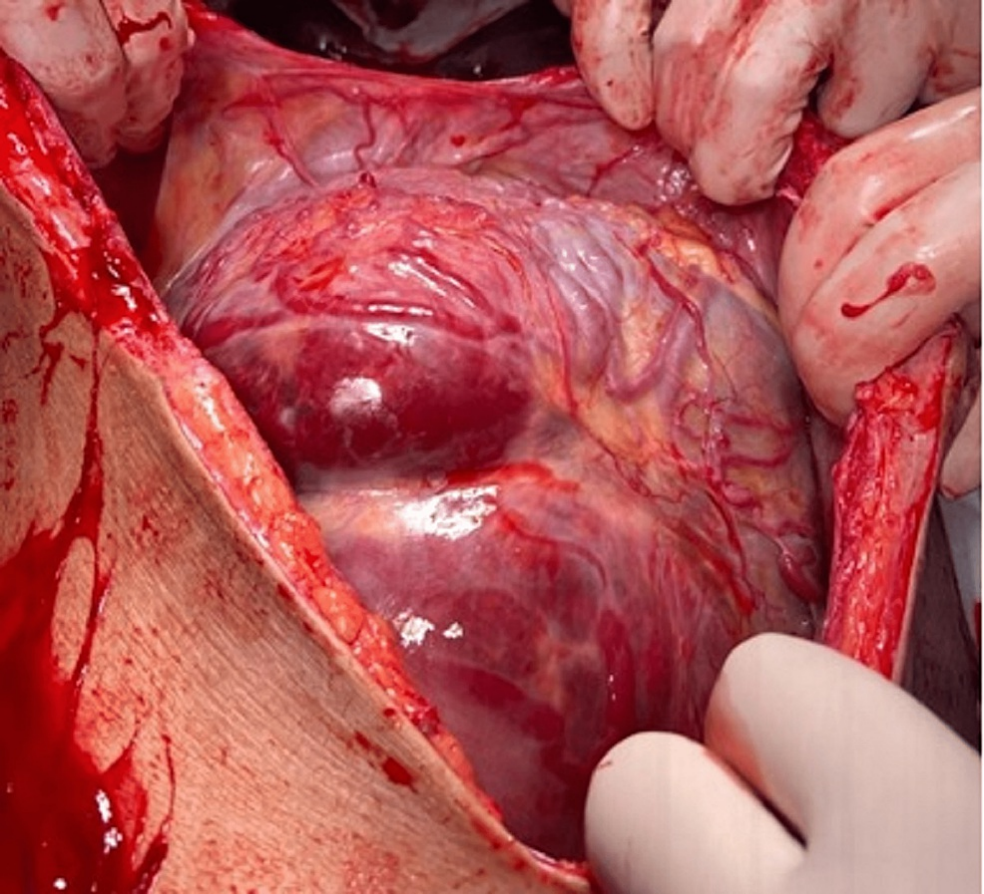
Placenta percreta invading the bladder.

During the intraoperative period, a placental bulge was seen invading beyond the uterus and involving the entire posterior wall and the bladder dome (Figure 1).

Therefore, a transverse fundal incision was given and the baby was delivered breech. Bilateral uterine arteries were ligated at their origin, and due to extensive involvement of the bladder wall, a decision for the placenta to be left in situ was made.

**Figure 2:**
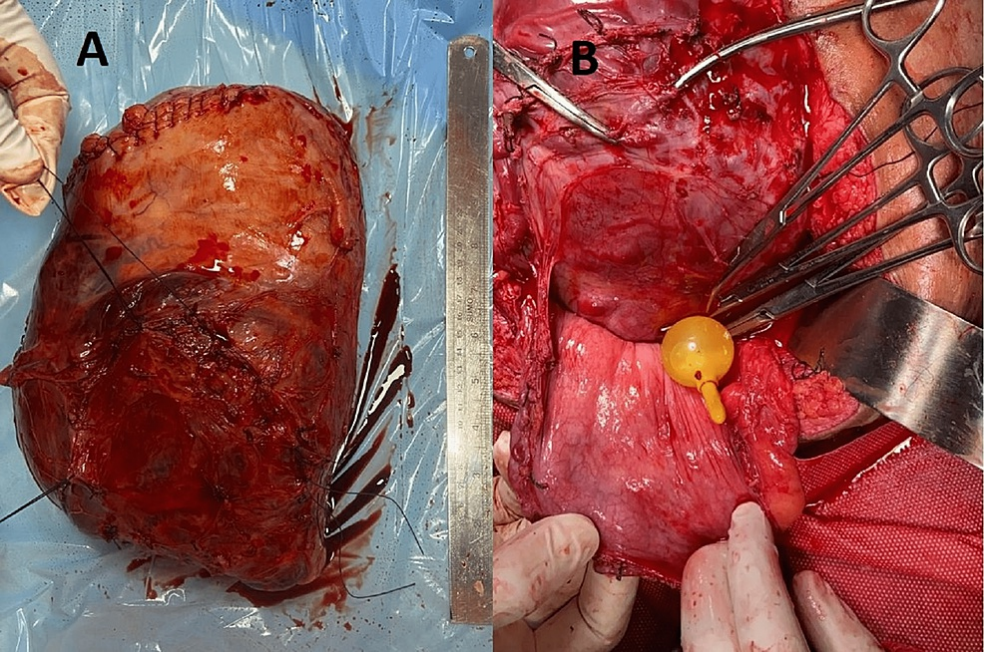
(A) Uterus with placenta percreta with resected bladder wall. (B) Resected posterior wall and dome of the bladder.

While shifting the patient to the ward she developed excessive vaginal bleeding due to which a decision for re-exploration was made, and the urology team was called for intraoperative assistance. The placenta was completely invading the bladder wall to the mucosa (Figure 2A). A cystotomy was made anteriorly and the bladder wall with areas of placental invasion was completely resected (Figure 2B). A hysterectomy was also done.

Both ureteral orifices were cannulated with 6-Fr feeding tubes. The bladder was closed in two layers after ensuring adequate hemostasis and removal of all parts of the placenta. Bilateral feeding tubes were exteriorized and a 16-Fr Foley catheter was placed. Feeding tubes were removed on postoperative day two, and a cystogram was performed after four weeks. The Foley catheter was removed after no evidence of contrast extravasation, and the patient voided well.

## Discussion

There are numerous risk factors for placenta percreta, as listed in Table 1 [5].

**Table 1:** Predisposing factors for placenta percreta

Factors
The major predisposing factor is the presence of placenta previa (a low-lying placenta) following cesarean birth
Maternal age of 33 years or older
Multiple cesarean births
Submucous myoma
Past curettage
Asherman’s syndrome
Smoking
Chronic hypertension
Prior history of adherent placenta problems

Clinical problems are related to the invasion of nearby or adjacent organs. Hematuria is an uncommon complication in 25% of all placenta percreta cases. Multiparous women having a history of cesarean deliveries with placenta previa and hematuria should be evaluated for placenta percreta. Women with any of the above risk factors should undergo pelvic ultrasonography with a high index of suspicion for placenta accreta. During pregnancy, patients with placenta percreta may have dull, persistent lower abdominal discomfort. Vaginal bleeding has been recorded during placenta percreta presentation [3]. Placenta percreta can lead to morbid complications such as hydroureteronephrosis and genitourinary fistulae and can be fatal due to excessive hemorrhage [6].

The optimal time to diagnose adherent placenta problems is during the prenatal stage. Ultrasound scans can reveal low-attached gestational sacs attached to the scar of a previous cesarean section. At the scar site, there is little myometrium. Ultrasound examinations during subsequent trimesters may reveal several placental lakes, including a Swiss cheese appearance, thin myometrium, loss of decidua placental layer, and an irregular bladder-myometrium boundary​​​​​​ [7]. If ultrasonography is inconclusive, magnetic resonance imaging (MRI) employing gadolinium contrast can be performed. Cystoscopy can be performed to assess posterior bladder wall-related abnormalities [8].

There are two main therapeutic options, namely, conservative management and cesarean hysterectomy.

Conservative management

The placenta is left in place and supplementary therapies can be administered afterward. Various adjuvant treatment modalities have been described, including vascular embolization, dilatation and curettage, methotrexate administration, and loop resection [9]. The most successful conservative treatment options for placenta percreta are angiography-guided balloon occlusion and embolization. These two techniques are intended to prevent hemorrhage during delivery. Internal iliac artery balloon catheters are implanted before delivery. Temporary occlusion using a balloon followed by a cesarean section minimizes blood loss and prevents major hemorrhage. ​​​​​Methotrexate should be used cautiously as therapeutic assistance for placenta percreta as its efficacy is not fully studied [10,11].

Hysterectomy and the role of the urologist

There is a higher likelihood of hemorrhage during an emergency hysterectomy, posing a substantial difficulty. The majority of placenta percreta include bladder wall invasion. Due to the risk of significant blood loss, the bladder should not be separated from the placenta as there is a risk of severe hemorrhage [3]. In cases when it is impossible to separate the bladder from the lower uterine segment, the posterior bladder wall can be removed along with the placenta, as was done in this instance. Abbas et al. recommend an anterior bladder wall cystotomy to identify dissection planes and assess whether posterior bladder wall resection is required [1]. Placental separation should be attempted if there is no mucosal and limited serosal involvement. In all such cases, an anterior cystostomy should be performed to determine the amount of bladder involvement.

**Figure 3:**
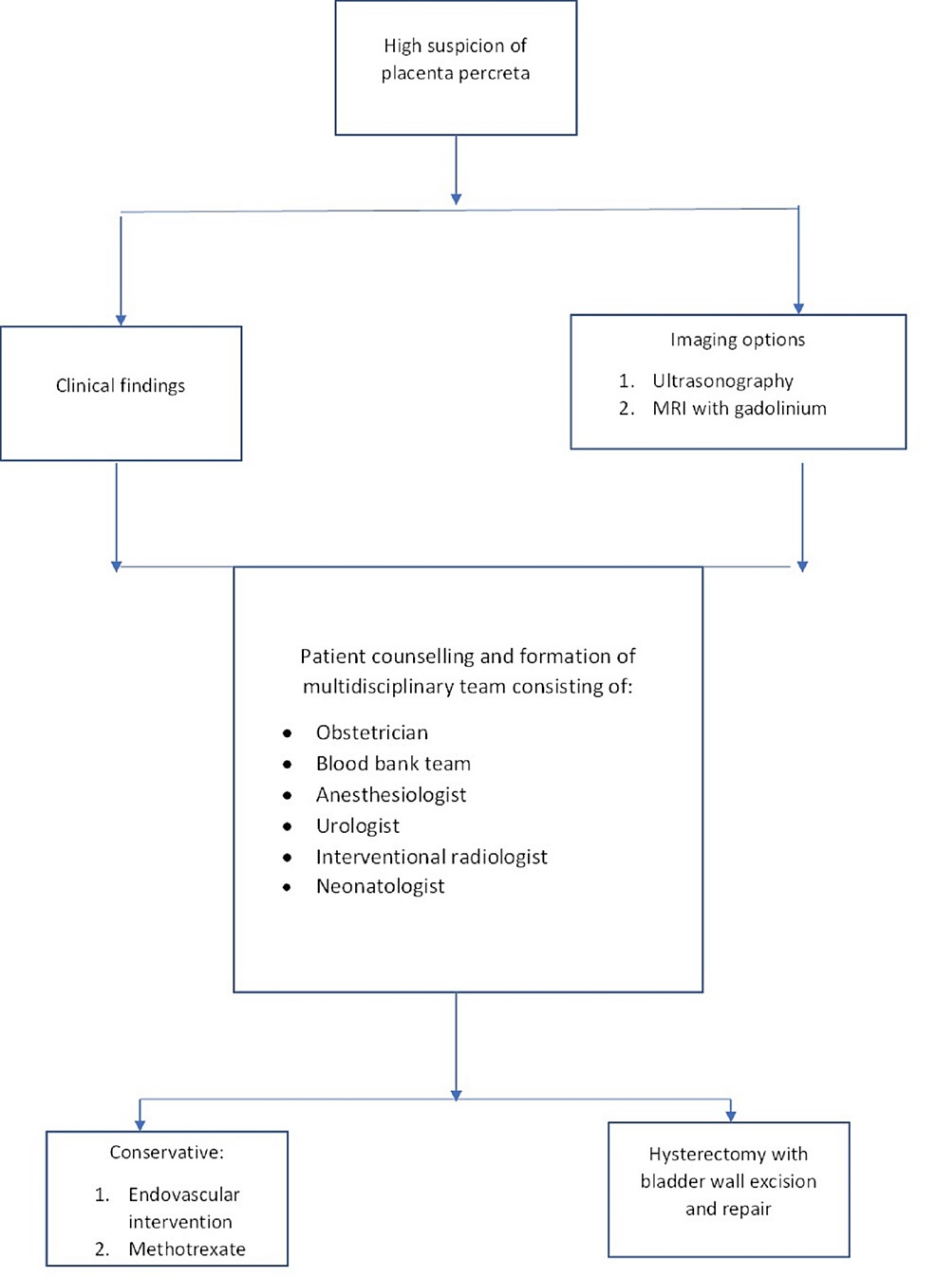
Approach for a patient with placenta percreta.

Incontinence and sexual dysfunction may be potential long-term outcomes of bladder injury. The trigone, detrusor muscle, and ureter orifices must be preserved. It is highly advised that a pelvic reconstruction-specialized urologist be present during the hysterectomy [3]. In situations of extreme blood loss, the surgeon must exercise discretion and take the necessary measures to save the bladder. Bladder cystotomy is advised because it can reveal the amount of the intruding placenta and assist the surgeon in selecting the ideal dissecting patterns for therapeutic effect [3]. Patients and family members should be informed of the increased risk of pregnancy-related deaths, the possibility of necessitating a hysterectomy, and the likelihood of bladder or ureter surgery. Figure 3 summarizes the approach for a patient with placenta percreta.

## Conclusions

Placenta percreta is a life-threatening condition complicated by various adjacent structural involvements and is often underdiagnosed preoperatively. Bladder involvement should be suspected in any case with hematuria episodes. The keys to successful management and better patient-related outcomes are a high index of suspicion and multidisciplinary management. Multidisciplinary management includes a urologist experienced in pelvic reconstructive surgery and a radiologist. The long-term consequences should be explained to the patient preoperatively to avoid any litigation.
